# Zika Fetal Neuropathogenesis: Etiology of a Viral Syndrome

**DOI:** 10.1371/journal.pntd.0004877

**Published:** 2016-08-25

**Authors:** Zachary A. Klase, Svetlana Khakhina, Adriano De Bernardi Schneider, Michael V. Callahan, Jill Glasspool-Malone, Robert Malone

**Affiliations:** 1 Department of Biological Sciences, University of the Sciences, Philadelphia, Pennsylvania, United States of America; 2 Department of Bioinformatics and Genomics, University of North Carolina at Charlotte, Charlotte, North Carolina, United States of America; 3 Department of Medicine, Division of Infectious Diseases, Massachusetts General Hospital, Boston, Massachusetts, United States of America; 4 Zika Foundation, College Station, Texas, United States of America; 5 Atheric Pharmaceutical, Scottsville, Virginia, United States of America; 6 Global Clinical Scholars Research Training Program, Harvard Medical School, Boston, Massachusetts, United States of America; Molecular Biology Unit (MBU), INDIA

## Abstract

The ongoing Zika virus epidemic in the Americas and the observed association with both fetal abnormalities (primary microcephaly) and adult autoimmune pathology (Guillain–Barré syndrome) has brought attention to this neglected pathogen. While initial case studies generated significant interest in the Zika virus outbreak, larger prospective epidemiology and basic virology studies examining the mechanisms of Zika viral infection and associated pathophysiology are only now starting to be published. In this review, we analyze Zika fetal neuropathogenesis from a comparative pathology perspective, using the historic metaphor of “TORCH” viral pathogenesis to provide context. By drawing parallels to other viral infections of the fetus, we identify common themes and mechanisms that may illuminate the observed pathology. The existing data on the susceptibility of various cells to both Zika and other flavivirus infections are summarized. Finally, we highlight relevant aspects of the known molecular mechanisms of flavivirus replication.

Zika virus (ZIKV), a mosquito-vectored flavivirus, was first isolated in 1947 from a sentinel research monkey caged in the Zika forest canopy within Uganda [1,2]. Soon after discovery, ZIKV was observed to infect humans [3]. Travel, shipping, and the worldwide distribution of human hosts and mosquito vectors (traditionally *Aedes aegypti* but likely other *Aedes* species and possibly *Culex* species [4–6]) has facilitated a global radiation of Zika viral infection [7]. More recently, introduction of ZIKV into naïve human populations has yielded rapidly spreading outbreaks in various Pacific island clusters (Cook Island, Easter Island, French Polynesia, and Micronesia), has resulted in the ongoing epidemic in the Americas (which may have originated in Haiti [8]), and has subsequently spread throughout Brazil, the Caribbean, and worldwide via travelers visiting affected regions [9,10]. In ZIKV-endemic regions such as continental Africa and Asia, there is epidemiologic support for the hypothesis that people are exposed to ZIKV during childhood and thereby develop immunity prior to puberty in both males and females. Introduction of ZIKV into dense, immunologically naïve populations has facilitated rapid viral evolution, including conserved modifications consistent with possible adaptation to a human host [11,12]. Most pertinent to the current concern about ZIKV is the infection of pregnant women who are immunologically naïve to ZIKV, intrauterine infection of their babies, and associated increased risk of congenital malformations consistent with other fetal pathogens such as those historically referred to by the TORCH acronym (Toxoplasmosis, Other [HIV, syphilis, varicella zoster virus (VZV), etc.], Rubella, Cytomegalovirus [CMV], and Herpes simplex virus-2 [HSV]).

ZIKV fetal syndrome resembles other intrauterine viral infections associated with congenital malformations but causes more severe abnormalities. Typical presentation of interpartum zika infection includes multiple defects: microcephaly, facial disproportionality, cutis gyrata, hypertonia and/or spasticity, hyperreflexia, and irritability. Abnormal neurologic image findings include coarse and anarchic calcifications mainly involving the subcortical cortical transition and the basal ganglia, ventriculomegaly secondary to the lack of brain tissue, and lissencephaly [10,13–16]. Genitourinary, cardiac, and digestive systems may also be affected [17]. This alarming and consistent clinical presentation provoked a rapid regional mobilization of public health experts in Pernambuco (in the Northeast Region of Brazil). Investigation soon revealed a correlation between ZIKV infection and the unusually high rate of infant microcephaly observed at the heart of the outbreak in Recife, Pernambuco. The striking features of ZIKV fetal syndrome may have gone unrecognized during prior outbreaks in the Pacific islands or may involve regional confounding variables or risk cofactors present in Brazil, such as prior exposure to dengue virus (DENV) [18,19], genomic changes in regionally circulating ZIKV [20–23], immunologic naivety and vaccination status of local populations [24,25], and exposure to pyriproxifen-containing insecticides [26] or thalidomide [27–30]. The current pathology may also be consequent to recent viral mutations, such as observed changes in the prM protein of the Brazilian ZIKV strains [11,31,32]. It has been demonstrated that ZIKV can infect human induced pluripotent stem cell–derived neural progenitor cells as well as human neurospheres and brain organoids in vitro, resulting in dysregulation of cell cycle–related pathways and increased cell death [33–36]. While the etiology remains unconfirmed, there appears to be a shift in the spectrum and incidence of birth defects between the latter stage of the French Polynesian outbreak [37] and what is now being observed in Recife, Rio, and throughout northern Brazil and surrounding regions [38,39]. In general, the combination of epidemiologic association and experimental research results strongly support a causal relationship between intrauterine ZIKV infection and fetal primary microcephaly.

Historically, human infection with ZIKV has presented in adults and young children as a mild, self-limiting, non-life threatening infection, with clinical symptoms appearing in 20% of infected patients and up to 80% being clinically asymptomatic during initial infection. Symptoms of acute ZIKV infection in otherwise immune-competent adults in the tropical Americas have clinical presentations similar to acutely infected patients in Southeast Asia who have been confirmed as Zika viral load–positive. When present, these symptoms typically persist an average of 4 to 5 days to approximately 1 week from initial onset of headache and fever. Key major symptoms following retro-orbital and frontal headache and fever include less consistent presentations of malaise, arthalgias, conjunctivitis, and pruritic maculopapular rash. More severe cases include escalation of the symptoms above as well as nausea, vomiting, and gastrointestinal distress [7]. The most recent assessment of clinical signs and symptoms of acute Zika virus infection observed in Puerto Rico includes rash (74%), myalgia (68%), headache (63%), fever (63%), arthralgia (63%), eye pain (51%), chills (50%), sore throat (34%), petechiae (31%), conjunctivitis (20%), nausea and/or vomiting (18%), and diarrhea (17%) [40]. Based on blood bank screens, viremia can begin up to 10 days before onset of symptoms [41], and the modest plasma viral titers observed often clear within 2 days of presentation with clinical symptoms, similar to what is observed with dengue [42]. At present, definitive diagnosis requires a polymerase chain reaction (PCR)-based test, and development of a rapid serologic diagnostic test is complicated by antibody cross-reactivity with other cocirculating arboviruses [43,44]. Historic serologic surveillance studies have been compromised by acute Zika infection induction of high titers of anti-dengue and even anti-chikungunya (CHIKV) convalescent immunoglobulin G (IgG) levels, routinely at titers above 1:1,280 [45,46]. Recently, the Centers for Disease Control and Prevention (CDC) has published guidelines for the testing of convalescent sera to rule out cross-reactivity by completing a time-intensive series of steps [47].

Current best estimates for the basic reproductive ratio (R_0_) for ZIKV varies between 1.2 and 6.6 [48–50], with a seroconversion rate approaching approximately 70% upon achieving maximal herd immunity. This limitation on further infection within a naïve population is typically achieved within 4 to 18 months of initial introduction [51,52]. Zika virus infection–associated acute motor axonal neuropathy-type Guillain–Barré syndrome (GBS) has been calculated to have occurred at a rate of approximately 1 in 5,000 cases of ZIKV during the outbreak in French Polynesia [19]; initial data for the rate of GBS and all combined neurologic disease in United States territories may be as high as 1% [40]. However, additional confirmation of these reports may show these numbers to be excessive. A clear temporal relationship between the peak of Zika virus infection in a susceptible population and a peak of GBS incidence 5 to 9 weeks later has been demonstrated, consistent with an autoimmune-mediated (rather than direct viral infectious neuropathy) pathologic mechanism [53]. Interim analysis of an ongoing prospective case study of ZIKV-infected pregnancies indicates a birth defect rate of circa 29% [38]. The lifetime cost for US care of an infant born with microcephaly has been estimated at 10 million in current (2016) US dollars [54]. In Texas between 2001 and 2010, 431,296 hospital stays were reported for children with birth defects, with total charges of US$24,800,000,000 [55]. For the sake of illustration, the potential impact of these epidemiologic estimates on the anticipated 2017–2018 Puerto Rico birth cohort is summarized in Fig 1.

**Fig 1:**
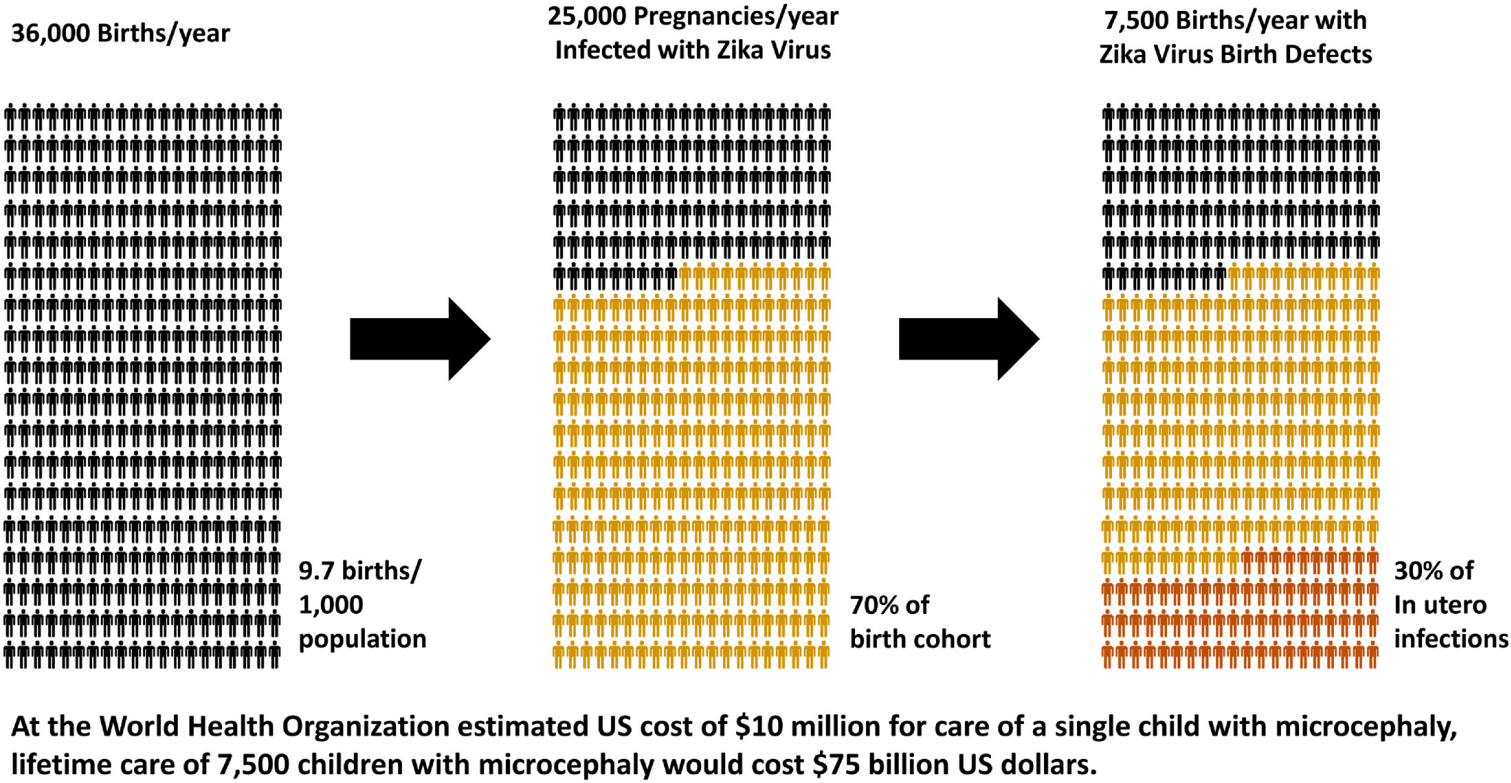
Projected teratogenic impact of maternal ZIKV infection on 2017–2018 birth cohort, Puerto Rico. For illustration purposes, the potential impact of unencumbered ZIKV spread through Puerto Rico on the cumulative one-year incidence of ZIKV-associated birth defects has been estimated and graphically summarized. Birth defect rate is based on preliminary data involving defects visible by in utero ultrasound examination from Brazilian (Rio) prospective pregnancy cohort study [38]. Final seroconversion rate of 70% is based on seroconversion observed with prior island outbreaks in Yap and French Polynesia [51,52]. Annual birth cohort for Puerto Rico is approximated as 36,000 infants, a number which presumes that the incidence of pregnancy is not impacted by anticipated risk of ZIKV infection or public health policy recommendations. Total birth defect rate associated with intrauterine ZIKV infection in Northern and Central Brazil is currently not determined and may exceed 30% of all Zika-infected pregnancies. WHO estimates of the US costs of caring for a single child with microcephaly are as high as $10 million [54]. 75 billion US dollars = US$75,000,000,000.

Zika virus has historically been considered to be vectored by *Aedes aegypti* mosquitoes. In contrast, *A*. *albopictus* is perceived to be the dominant vector of DENV, CHIKV and now ZIKV in temperate climes of Southern Brazil and Northern Argentina. Mosquito traps show no *A*. *aegypti* in these regions, but the regional ZIKV incidence of approximately 1:1,000 suggests that the cold-tolerant *A*. *albopictus* may be a major insect vector for ZIKV transmission above 2,000 meter elevations and in cooler climes. Zika virus sequences have been difficult to detect in trapped mosquitoes from outbreak areas but have recently been recovered from *A*. *albopictus* mosquitoes by the laboratory of the Institute of Epidemiological Diagnosis and Reference (InDRE), which functions as part of Mexico’s National Epidemiological Surveillance System (SINAVE) [56]. Identifying the virus in wild-caught mosquitoes is not a simple task. However, reports are starting to circulate describing the isolation of ZIKV from or the growth of the virus in *Culex* mosquitoes [5]. The expanded geographic range and behavior of this new potential vector must be considered when considering ZIKV prevention.

The current outbreak has highlighted other routes of transmission, including person to person spread. Infective ZIKV has been isolated from urine and saliva of patients in Brazil [57]. ZIKV is more stable than DENV [31], and so it cannot be assumed that sexual transmission is the only means of direct human-to-human infection. In the current outbreak in the Americas, there is strong evidence for frequent sexual transmission of the virus, including from otherwise asymptomatic individuals [58–62]. As only one in five ZIKV-infected individuals develop clinical symptoms, sexual transmission from an otherwise asymptomatic infected male to his partner [62] suggests the possibility of large numbers of occult-infected males in endemic regions who are unknowingly transmitting ZIKV to their partners. Furthermore, the risk of sustained viral loads in males returning from ZIKV-endemic regions to foreign nations with temperate climates and endemic *Aedes* and *Culex* mosquitoes poses a theoretical risk that male travelers may expand the range of sylvatic transmission in regions with capable *Aedes* (and perhaps *Culex*) vectors. While ZIKV RNA can be detected in breast milk, urine, semen, and sputum from infected individuals [63], replication-competent virions have been most readily cultured from semen samples. Semen ZIKV RNA levels may be up to 100,000 times higher than corresponding plasma levels [64]. Preferential ZIKV replication in testes has been hypothesized. ZIKV is shed in semen for an extended period, and the average duration of shedding has yet to be determined [64]. The stability of ZIKV in aqueous suspension, on surfaces, or as fomites is unknown, but other flaviviruses can persist under various ambient conditions for extended periods [65–69]. The presence of infectious ZIKV particles in semen, urine, and saliva of patients during the acute phase may play a critical role in the spread of ZIKV within infected regions. The relative contribution of alternative nonvectorial (direct human-to-human) ZIKV transmission routes needs further investigation, particularly in light of the increased stability of ZIKV relative to other flaviviruses [57].

Sequence comparisons of ZIKV isolates indicate significant genetic differences between historic samples obtained from mosquito species and more modern isolates from human sources, including human samples obtained during the current outbreak in the Americas [11,12]. Any clinical significance associated with these viral genetic changes has yet to be elucidated, although studies in murine models and with human neurospheres supports the hypothesis that the ZIKV strain circulating in the Americas is neuropathic and more teratogenic than an African isolate [70].

The apparent teratogenic effects of ZIKV infection have turned what was once considered a relatively benign pathogen into a subject of great social and scientific concern. Detection of ZIKV RNA and particles in amniotic fluid and fetal brain obtained from the products of conception strongly suggest that the virus is capable of directly infecting fetal tissue [15,16]. When considering the vast array of human pathogens, the probability of a mother passing an infection to her developing fetus is relatively rare. However, examples of pathogens consistently capable of vertical intrauterine transmission do exist and can be associated with teratogenic effects. These viral diseases involving intrauterine infection may illuminate and inform research into the possible mechanisms by which ZIKV may induce fetal neuropathology, as well as other birth defects, and may facilitate development of public health risk mitigation strategies and potential treatments.

## TORCH Viral Pathogens

Teratogenic infectious agents that are vertically transmitted from mother to infant during pregnancy, childbirth, or breastfeeding have traditionally been classified as TORCH pathogens. For the purpose of this review, we will focus on the classical viral TORCH pathogens: rubella, cytomegalovirus, herpes simplex virus, and varicella zoster virus. These viruses can cross the placenta and cause congenital defects including, but not limited to, microcephaly, growth and mental retardation, heart disease, hearing loss, and blindness [71–73]. Years of scientific research concerning TORCH pathogen infection and teratogenicity have yet to identify therapeutic interventions that reduce occurrence of serious medical sequela and miscarriages for most of these viruses. Current preventative measures are limited to vaccination and avoiding viral exposure or the use of acyclovir for HSV infections [74]. These approaches have limitations and are not globally available. The most extensive fetal damage associated with viral TORCH infections typically takes place when the mother is infected during first eight weeks of the pregnancy, during which time the central nervous system (CNS) of the developing fetus is actively forming. With most viral TORCH pathogens, birth defect risk and severity is significantly reduced when infection occurs after seventeen weeks of gestation [75]. Often, first trimester infections result in miscarriages. Not all fetal congenital abnormalities manifest clinically at birth and may present later in a child’s development. As summarized in Table 1, presence of congenital defects at birth is typically linked to TORCH infection at earlier stages of gestation.

**Table 1:** Selected viral TORCH pathogens and associated morbidity. After [75].

Viral TORCH Pathogen	Symptoms	First or Second Trimester Teratogen	Third Trimester Teratogen	Primary microcephaly	Spontaneous abortion or fetal death
**Rubella virus (German measles)**	Defects in multiple organ systems, including the ophthalmic (cataracts and microphthalmia), cardiac, and neurological (deafness, mental retardation), and increased risk of type 1 diabetes in childhood	+	-	+	+
**Cytomegalovirus**	Mental retardation, sensorineural hearing loss, jaundice, hepatosplenomegaly, petechiae, preterm birth, preeclampsia, and fetal growth restriction	+	-	+	+
**Herpes simplex virus**	Encephalitis, sepsis, cataracts, pneumonitis, myocarditis, hepatosplenomegaly, chorioretinitis, and mental retardation	+	+	+	+
**Varicella zoster virus (chickenpox)**	Skin lesions, neurological and eye defects, limb hypoplasia, fetal growth restriction, and defects of multiple organ systems	+	-	+/-	+
**Zika virus**	Microcephaly, facial disproportionality, cutis gyrata, hypertonia and/or spasticity, hyperreflexia, and irritability; abnormal neuroimages include calcifications, ventriculomegaly, and lissencephaly	+	+	+	+

### Rubella (German Measles)

Prior epidemic outbreaks of rubella and consequent associated birth defects may provide the best illustration of the neonatal health risks of the current ZIKV outbreak in the Americas [76], although the incidence of congenital rubella syndrome (CRS) associated with initial outbreaks in rubella-naïve populations [77] appears to have been significantly less than what is being documented with ZIKV in Brazil [38]. Rubella virus (RuV) is a member of the *Rubivirus* genus and Togaviridae family. The rubella genome is encoded on a positive single-stranded RNA (ssRNA), which is assembled on a protein scaffold and surrounded by a lipid envelope. Host cell infection with RuV is driven by two glycoproteins, E1 and E2. Encoded in by the RuV genome, these glycoproteins assemble as heterodimers on the surface of the viral envelope and function similarly to the fusion proteins of flaviviruses [78,79]. E1 protein trimer directly inserts into the host cell plasma membrane lipid bilayer and, using a hairpin motion, brings the RuV closer to the cell surface to facilitate endocytosis [80,81]. The release of the viral genome into the host cell occurs via low pH and with the Ca^2+^-dependent E1 trimer conformational changes associated with maturing endocytic vesicles [82]. Recent work has identified myelin oligodendrocyte glycoprotein as a receptor with affinity for RuV E1 protein [83]. This discovery may provide a causal link between rubella virus and brain damage in fetuses with CRS, as use of this receptor by the virus may enable infection of oligodendrocytes in the developing brain. RuV infection of pregnant women has a pronounced teratogenic effect, especially during the first gestational trimester [80]. Pathological and immunohistochemical analyses of aborted fetuses with CRS demonstrated widespread necrosis to organs—including the eye, heart, brain, and ear—and are associated with the presence of rubella virus in all tissues [84]. In vitro studies suggest that RuV infection inhibits normal growth and differentiation of human embryonic mesenchymal cells [85]. RuV-encoded replicase P90 protein has been shown to disrupt actin cytoskeleton formation by directly binding and inhibiting Cytron-K kinase, a cytokinesis regulatory protein [86]. Inhibition of Cytron-K leads to cell cycle arrest and apoptosis in developing neuronal populations and retina of in vitro cultured mouse embryos [87]. Additionally, rubella virus infection of placenta and embryonic cells induces interferon expression, especially in the placenta [88]. This ability to infect and alter the placenta likely allows the virus access to the rest of the fetus. The most commonly observed outcomes of CRS are congenital cataracts (97.4%), inner ear abnormalities (73.9%), microcephaly (68.4%), and congenital heart defects (57.9%) [73,84,89,90]. If the infection occurs during the first trimester, the rate of CRS is 80%–90%. Odds of intrauterine development of extensive CRS dramatically decreases after 12 weeks of gestation [71].

### Cytomegalovirus (CMV)

CMV is a member of the Herpesviridae family, Betaherpesvirinae subfamily, and is also known as human herpesvirus 5 (HHV-5). Intrauterine CMV infection is linked to development of severe neurological handicaps, microcephaly (36%), intracranial calcifications, microgyria, eye defects, and sensorineural hearing loss [89,91–93]. Congenital CMV infections are associated with radiographic findings that vary with gestational age at time of infection. Lissencephaly, including thin cerebral cortices, extremely diminished volume of white matter, delayed myelination, small cerebella, and very enlarged lateral ventricles have been correlated with CMV infection prior to 18 weeks of gestational age, whereas those cases of congenital CMV infection, which present with more normal gyral patterns (normal cerebral cortices, slightly diminished volume of white matter, delayed myelination, normal cerebella, and slightly enlarged lateral ventricles), are associated with third trimester infection [94,95]. These findings are similar to those observed with heritable disorders, including cystic leukoencephalopathy without megalencephaly, Aicardi–Goutières syndrome, type 1 interferonopathies, and RNASET2-related leukodystrophy [96,97].

CMV is a double-stranded DNA virus (dsDNA) with a complex envelope structure of 12 glycoproteins. Because of this complexity, CMV can bind to a broad spectrum of cell surface receptors and quickly becomes ubiquitous in the human host after initial infection [98]. CMV glycoprotein gB and heterodimer gM/gN have affinity to heparan sulfate proteoglycans (HSPGs), which are abundantly present on the surface of most cell types [99,100]. Additionally, CMV has been shown to bind epidermal growth factor receptor (EGFR) and β1 integrin co-receptors, thereby facilitating proximity to the host cell membrane [101,102].

CMV crosses host cell barriers via membrane fusion mediated by the gH/gL/gO and gB viral envelope glycoproteins [101,103]. CMV infection is mostly asymptomatic in immune-competent adults, and forms a lifelong latent infection. Primary CMV infection during pregnancy yields the highest risk of vertical transmission (32%) relative to virus reactivation in chronically infected mothers (1.4%) [104]. CMV infection of the cytotrophoblast progenitor cells associated with floating villi in the placenta appears to elicit a shift in the Th1-type cytokine and Th2-type cytokine (Th1/Th2) ratio balance of amniotic fluid and placental tissues towards a Th1 profile by upregulation of proinflammatory cytokines like monocyte chemoattractant protein-1 (MCP-1) and tumor necrosis factor (TNF) [105,106]. This shift has been hypothesized to directly induce defects in placental formation and congenital abnormalities. There is significant evidence supporting the hypothesis that CMV virions transit placental barriers to fetal infection by co-opting the neonatal Fc receptor–mediated transport pathway for IgG (transcytosis) [107]. However, replication of CMV in uterine endothelial cells may be required for subsequent infection of cytotrophoblasts [108,109].

### HSV (HSV-1 and HSV-2)

HSV is a dsDNA enveloped virus belonging to the Herpesviridae family. Similar to CMV, HSV has a large number of glycoproteins present on the surface of its viral envelope and can bind to multiple host cell receptors [110]. HSV infection leads to formation of oral (HSV-1) and genital (HSV-2) lesions in adults. HSV host cell entry requires a viral glycoprotein, primarily glycoprotein D (gD) binding to heparan sulfate and HveA (Herpes Virus Entry Mediator [HVEM] receptor), HveB (nectin-2), or HveC (nectin-1) receptors on the host cell plasma membrane surface. HSV enters the host cell via membrane fusion or endocytosis [110]. HSV can enter the CNS of adults and in rare cases has been associated with clinical encephalitis [111]. HSV infects neuronal cells through the nectine-1 receptor and can form a latent and immunologically privileged reservoir of infection in the brain [112].

In contrast to CMV, cross-placental transition of HSV from mother to fetus is uncommon [113]. Cells of the outer layer of the placenta do not express HveA, HveB, or HveC and cannot be infected by HSV [114]. Congenital HSV infection is very rare and usually occurs when a serologically negative mother is exposed to the virus during the first trimester of pregnancy. Congenital HSV pathology includes multiorgan failure, liver necrosis, encephalitis, microcephaly (32%), hydrocephalus, chorioretinitis, and skin lesions [115,116]. HSV infection of placenta-associated cells induces inflammation and necrosis of placental tissue [115]. Neonatal HSV-2 infection during childbirth or HSV-1 infection during the first year of life is more common and is associated with up to 40% mortality. Aggressive anti-HSV treatment of neonates with acyclovir often controls the virus at the cost of long-lasting health risks to the child [117]. There is a higher risk for HSV infection of the infant during childbirth in mothers that acquired genital HSV during the last trimester (~50%), while peripartum HSV-2 reactivation is associated with less than 1% of neonatal infections [117]. This result suggests the role of maternal antibodies in protection of the child from HSV infection during birth. Congenital HSV infection is differentiated from perinatal infection by early onset (within 24h of birth) and increased severity of the symptoms [71]. The relatively rare event of HSV microcephaly is exclusively associated with congenital infections [116].

### VZV (Chickenpox)

Varicella zoster virus is a dsDNA enveloped virus. It belongs to Herpesviridae family and Alphaherpesvirinae subfamily. VZV and HSV belong to the same subfamily and share many characteristics [118]. Similar to HSV, VZV can cause encephalitis and can also form latent viral reservoirs in the brain [111,119]. The VSV viral envelope glycoprotein gE is essential for infection. This protein binds the insulin-degrading enzyme (IDE) receptor and employs heparan sulfate to facilitate host cell infection [120]. Congenital VZV is associated with a high neonatal mortality rate (30%). Primary VZV infection during the first 6 months of pregnancy is associated with a 25% risk of in utero infection [72]. Twelve percent of intrauterine infections will result in a range of birth defects, including limb hypoplasia, microcephaly, hydrocephaly, mental retardation, and cataracts [72], in many ways similar to the disease spectrum currently observed with Zika congenital syndrome.

## Zika Virus: A New Viral TORCH Pathogen

The list of TORCH viral pathogens is constantly expanding, and sufficient clinical data support adding ZIKV to the list. The exposure of a naïve population to a new virus which has historically been mosquito-vectored, is sexually transmissible, and may be capable of direct human-to-human transmission by other means presents a greater challenge. With the emerging global threat of ZIKV infection to pregnant women, it is critical that we improve our understanding of the mechanism(s) of intrauterine infection and of the medical management of subsequent neurologic disease.

Examination of the classic TORCH pathogens reveals some common themes that can inform research concerning ZIKV fetal neuropathogenesis: these agents either infect the placenta or infect specific tissues in the fetus linked to pathology. For example, rubella can infect the placenta and CNS tissue; CMV infects and damages the placenta; HSV infects cells proximal to the placenta, leading to necrosis; and HSV as well as VZV are capable of infecting neurons. In some cases, specific molecular mechanisms that exacerbate the resulting pathology have been identified. Further exploration of cell surface receptors and placental permeability may assist with development of interventional prophylactics and therapeutics for pregnant women.

### Zika Virus Infection of the Placenta and Fetal Brain

In order to successfully establish an infection in a target tissue, all viruses must go through the same basic steps: the virus must overcome local host defenses at the site of infection (both barrier and immunologic response), infect a cell that is both susceptible and permissive to producing infectious virions, and the infected cell must release sufficient numbers of infectious particles that are able to travel to the target tissue and again infect a susceptible cell. Analyzing what we know about ZIKV infection in terms of this model can shed light on the possible mechanisms by which ZIKV might cause fetal abnormalities after initial maternal infection.

There are many plausible alternative hypotheses for Zika virus-induced fetal neuropathogenesis [121]. These alternatives generally fall into two categories: infection of fetal tissue by ZIKV or transcytosis of other factors that are causative of Zika congenital syndrome. Infection of fetal tissue may involve transcytosis of ZIKV from mother across the placenta or infection of the placenta itself. Either option may lead to dissemination of the virus in the fetus and subsequent infection of the developing brain. Infection of the placenta and resulting inflammatory response may indirectly alter neural development. Transcytosis of (yet to be defined) antigen-specific immunoglobulins or other maternal molecules related to the development of ZIKV GBS may directly harm the fetal brain without requiring viral replication in nervous tissue [19,122,123]. ZIKV transfer and infection of the developing fetal brain may occur directly as free virions, as virion non-neutralizing antibody complexes [124], or via infected Hofbauer or other migratory cells [125,126]. Activation of TLR (Toll-like receptor)-3 by ZIKV binding to nervous tissue cells may directly induce damage without requiring viral replication [36]. Placental infection by ZIKV—triggering induction and release of inflammatory response–associated molecules—may be sufficient to indirectly damage the fetal CNS [127–129]. These possible mechanisms are not mutually exclusive and may operate at different stages of fetal development.

The placenta represents a major barrier to fetal infection. This organ has evolved pathways for regulating the transport of materials, metabolites, oxygen, and electrolytes as well as both innate and adaptive immunologic effectors (particularly maternal immunoglobulin) between the mother and fetus. Soluble factors, oxygen, and cells can all be selectively exchanged. Despite the relatively common event of infection of a pregnant woman by different viruses, transplacental passage of virus and intrauterine fetal infections are rare. This high degree of selectivity is largely due to a specialized outer placental layer: the syncytiotrophoblast, a large, multinuclear body formed by the fusion of multiple cells into a syncytium during the second trimester of fetal development [130]. This fusion into a single giant cell avoids the problems of maintaining intercellular junctions, which are sufficiently tight to prevent the unregulated movement of large molecules (and pathogens). In order for a virus to reach the fetus after this event, ZIKV must either have a mechanism to bypass the syncytiotrophoblast barrier or must directly infect the placenta itself, as has been observed with various viral TORCH pathogens. One possible method for the passage of ZIKV across the placenta to the fetus is through the mechanism that facilitates unidirectional transmission of maternal antibodies to the amniotic fluid and developing embryo [131,132]. The neonatal Fc receptor (FcRn or FCGRT) is proposed to be involved in the recognition of maternal IgG and in uptake of these antibodies by the cells of the infant gut. In addition, neonatal Fc gamma receptor IIb2 molecules expressed in human villous endothelium (within the FCGR2B2 compartment) actively participate in endothelial transcytosis of maternal IgG [133,134]. RAB3D, a member of the RAS-related protein Rab family, appears to play a key role in regulating the activity of the FCGR2B2 organelle and therefore may influence transport of either autoimmune-associated antibodies or antibody-coated ZIKV. Antibody mediated enhancement of infection has been reported for dengue virus, a related flavivirus, as well as for ZIKV [18,124]. For dengue virus, antibodies raised against previous infection with a different serotype of virus may enhance subsequent infection in a dendritic cell–mediated fashion [135,136]. For ZIKV, in vitro studies have demonstrated enhancement of infectivity with serum from patients with serologic responses to dengue virus [18,124]. The high degree of cross-reactivity between antibodies elicited by co-circulating arboviruses present in Brazil and throughout the Caribbean may contribute to intrauterine ZIKV disease by facilitating infected dendritic cell transport or by direct transcytosis of non-neutralizing, antibody-coated ZIKV virions [18].

Delivery of ZIKV by transcytosis of antibody-bound virions does not appear to be compatible with the window of greatest vulnerability for Zika teratogenicity: the first trimester of pregnancy. The transport of maternal IgG across the placenta begins at week 16 [137,138]; the levels of IgG in fetal circulation at gestational weeks 17–22 are relatively low (5%–10% of maternal levels) and rise continually, with levels reaching 50% at weeks 28–32, followed by an exponential increase in the final 4 weeks before delivery [139]. A study of RNA levels of Fc receptors in the placenta confirms that transcytosis is likely to begin primarily in the second trimester [140]. Functionally active placental FcRn expression has been detected at 20 weeks [141]. By analogy, maternal autoimmune antibodies, which may be elicited by ZIKV epitope mimics (ergo, GBS-associated antibodies) [142] are also unlikely to cross the placenta prior to the 16th gestational week. Many mothers of microcephalic children were infected with ZIKV before the 10th gestational week and were likely to have cleared the virus well before 16 weeks [44]. Relevant to this observation, fetal cerebrospinal fluid (CSF) levels of IgG are higher than what is seen in newborns and adults [143]. This suggests that an incompletely formed fetal blood–brain barrier may allow concentration of IgG in the developing CNS. This observation may explain some of the observed specificity for ZIKV infection of fetal brain in contrast to other tissues. Antibody-mediated enhancement may still have a role to play in the infection of the developing brain, secondary to the virus overcoming the placental barrier. This theory fits well with observations that the fetal blood–brain barrier actively excludes IgG in the third trimester, a period when maternal infection has been associated with less fatal neuropathogenesis.

The timing of ZIKV infection relative to neonatal outcome may illuminate the mechanism of fetal infection. A recent preliminary report describes neuropathological aspects of fetal development in a cohort of Zika-infected women [38]. Most strikingly, fetal ultrasonography revealed abnormalities in 12 of the 42 women who experienced ZIKV infection during pregnancy, as compared to none of the 16 cohort-matched fetuses in Zika-negative women. Although the size of the cohort studied in this reported in this study was still low, they span a period of initial ZIKV exposure running from 8 weeks to 35 weeks of gestation. The observations of microcephaly and severe cerebral pathology appear most commonly when the mother was infected with ZIKV at 12 weeks or earlier. Infection of the mother during the second or third trimester was reported to result in intrauterine growth restriction or, in two cases, fetal death. This pattern of timing supports the hypothesis that first trimester infection results in direct transmission of the virus to the fetal brain with subsequent viral replication, whereas later infection may involve activation of placental inflammatory responses. ZIKV infection of human cerebral organoids acts (at least in part) via TLR-3 to elicit a direct neural cell depletion, which is partially abrogated by TLR-3 inhibition. TLR-3 activation by ZIKV resulted in alterations in expression of multiple genes associated with neuronal development, implying a mechanistic connection to disrupted neurogenesis [36].

The overall retardation of growth observed after second and third trimester exposure to ZIKV suggests that the virus may be exerting an indirect teratogenic effect by infecting the placenta rather than other fetal tissues during this period. A separate case study has identified infectious virus in the placenta of a fetus and detected resulting ongoing maternal ZIKV viremia [15], and this may include placental Hofbauer cell infection and/or activation [125,126,129]. This is in agreement with previously published work showing that the placenta can induce viral resistance in nearby cells [144]. In contrast, a well-designed basic virology study has shown that placental cells from a full-term pregnancy are resistant to ZIKV [145]. However, no data currently exist concerning the susceptibility of early placental cells to ZIKV infection.

Another possible mode of fetal infection would be transmission of ZIKV-infected maternal cells across the placenta at any stage of pregnancy. If a motile cell (such as a dendritic or Hofbauer cell) was infected and then crossed the placenta or was able to transit maternal-placental blood vessels, it could carry virus to the fetus [125,126]. A similar situation has been modeled in mice, in which dendritic cells can carry intracellular pathogens across the placenta [146]. There is some limited evidence for the presence of maternal cells in the lymph nodes of second trimester fetuses, but the mechanism by which this migration occurs is not well understood [147]. Infected migratory maternal cells might also contribute to fetal neuropathology via proinflammatory cytokine release. Placental Hofbauer cells have been shown to be activated by TLR-3 and TLR-4–mediated pathways, and ZIKV has been shown to activate TLR-3–mediated responses in neuronal cells [36].

Teratogenicity and neuropathology associated with TORCH pathogen infection of the placenta is well documented [75], and ZIKV may also interfere with fetal development by this route [127]. The pronounced elevation of a variety of inflammatory cytokines may trigger microglial activation—with attendant damage to surrounding cells, including neurophils—but is usually associated with damage to a wide range of fetal organs and tissue [148]. The disease spectrum associated with chorioamnionitis overlaps with many of the features of Zika congenital syndrome and includes periventricular leukomalacia, intraventricular hemorrhage, cerebral palsy, and retinopathy of prematurity [149–153]. While ZIKV may also elicit similar pathology by direct placental infection, the striking selectivity and consistency of central nervous system damage observed, combined with the unusually severe damage to developing brain and the presence of ZIKV sequences in amniotic fluid and brain tissue, suggests some contribution of direct ZIKV infection of fetal CNS in the majority of cases.

The development of several mouse models for the study of ZIKV infection has shed light on some of the questions regarding the involvement of the placenta in fetal infection. Three distinct mouse models have recently been developed for the study of ZIKV. In the first model, isolated ZIKV is injected directly into the cerebroventricular space and/or lateral ventricle of embryonic day 13.5 fetal mice of the ICR strain [154]. The second involves the infection of mice that lack competent interferon signaling interferon-α/β receptor (Ifnar -/-, A129, or AG129 strains) [155]. This model leads to high viral loads in the brain, spinal cord, and testes and recapitulates some of the more severe neuropathogenesis seen in humans. Infection of Ifnar-/- mice has also been applied to the study of fetal pathogenesis [156,157]. The third model involves the infection of immunocompetent SJL mice with ZIKV during pregnancy [70]. Because of the ability to infect the mother and observe transmission to the fetus, the second and third models are useful in evaluating the involvement of placenta in Zika congenital syndrome. Miner et al. make use of Ifnar-/- mice to study the effect of ZIKV infection of the mother on the placenta [156]. In this particular model, no isolated microcephaly is observed. However, the ZIKV can be detected in the fetal CNS, and the fetuses do show signs of intrauterine growth restriction. Examination of the placenta reveals robust infection of various trophoblast cells. This was associated with damage to the placental blood vessels and an overall smaller placental size. Treatment of wild-type mice with anti-interferon antibodies allowed similar infection of the placenta and fetal CNS to occur. This highlights a role for interferon in protecting the placenta from ZIKV infection and fits well with the description of term placental cells being protected from infection because of interferon response [158]. Dengue virus was unable to infect the placenta or fetus in this model. These findings confirm that ZIKV is relatively unique amongst the flaviviruses in being a TORCH pathogen, although infrequently observed clinical human congenital infection by the closely related West Nile virus (WNV) indicates that virus is also associated with a variety of birth defects including aortic coarctation, cleft palate, Down syndrome, lissencephaly, microcephaly, polydactyly, and abnormal growth [159]. The study by Cugola et al. primarily employs the SJL strain of mice but does examine C57BL/6 mouse infections and finds no fetal pathogenesis in this strain. This strongly suggests that genetic differences may control the ability of the Zika virus to cross the placenta. Identifying why some strains allow placental infection while others do not will be crucial in determining how this mechanism functions in humans.

### Expression of ZIKV Receptors in Placental and Central Nervous System Tissues

Early in embryonic development, direct infection of the placenta by ZIKV could provide a route of entry to fetal tissue. Productive infection of the trophoblast by the virus would allow newly produced virions to be passed inward to the fetus. A critical step to the productive infection of any target cell is the expression of the correct viral receptors on the cell surface.

Flaviviruses, such as dengue virus, Japanese encephalitis virus (JEV), and West Nile virus (WNV), are known to use cellular C-type lectin proteins as receptors [160]. Expression of several members of this receptor family is high on cells of the myeloid lineage, such as monocytes, macrophages, and dendritic cells [161]. Multiple studies provide evidence for the role of one specific lectin, dendritic cell–specific ICAM-3–grabbing nonintegrin (DC-SIGN), in the infection of flaviviruses [162–166]. DC-SIGN is an essential host protein that is involved in pathogen capture and antigen presentation in dendritic cells. As a lectin, DC-SIGN recognizes carbohydrate structures on proteins. Any ZIKV transmitted to a human host after replication in the salivary gland of a mosquito vector will carry the glycosylation pattern produced in the cells of the insect host. When the virus replicates in insect salivary glands, the glycosylation of the viral proteins involved in receptor binding will follow the pattern observed in insects (high-mannose glycans) and not the more complex pattern seen in mammalian glycoproteins [163,167]. Dendritic cells are capable of recognizing this difference and reacting to these nonhost glycosylation patterns. This specificity and the presence of dendritic cells in the epidermis (and therefore in close proximity to the site of the mosquito bite) means that mosquito-vectored flaviviruses are likely to preferentially infect the dendritic cell as an initial target cell type. The probability of uptake and initial infection of host dendritic cells may be enhanced by the presence of preexisting non-neutralizing antibody that binds ZIKV [18].

Although the initial stages of human ZIKV infection are not as extensively studied as infection with viruses such as dengue, a study by Hamel et al. has identified multiple receptors involved in ZIKV entry to the target cell [168]. This seminal work examined the involvement of known dengue virus receptors in ZIKV infection. The results confirmed a role for DC-SIGN in mediating ZIKV entry and also identified roles for two TAM receptor proteins called Tyro3 and AXL and a minor role for a protein called T-cell immunoglobulin and mucin domain 1 TIM-1. Tyro3 and AXL are tyrosine kinase receptors whose natural ligand are the vitamin K–dependent proteins growth arrest–specific gene 6 (Gas6) and protein S. Armed with this list of receptors, it is possible to predict what specific cells in the placenta and CNS might be susceptible to ZIKV infection.

An analysis from the US Centers for Disease Control and Prevention (CDC) reported ZIKV RNA and proteins in tissues from newborns and from two miscarriages [169]. Examination of the corresponding placentas showed pathology associated with viral infection. Direct ZIKV infection of the placenta is plausible, as the trophoblast layer has been shown to express the needed receptors, and a recent report has recovered infectious virus from the placenta [15]. AXL expression has been detected in the trophoblast, and perturbations in Gas6 signaling through AXL have been shown to be associated with preeclampsia, suggesting a possible mechanism of pathology [170]. Histology available through the Human Protein Atlas also confirms expression of AXL and Tyro3 throughout the trophoblast layer [171]. Although the trophoblast does not appear to express DC-SIGN, tissue-resident cells of the myeloid lineage will express this lectin. This provides a pathway by which the infected trophoblast might produce a virus that will infect patrolling myeloid cells. Infected myeloid cells may allow production of greater quantities of the virus (leading to viremia) or serve as a vector to traffic the virus to other tissues. Proof of this second possibility requires the identification of ZIKV-positive perivascular macrophages or microglia in brain tissue from abortus specimens.

In order to selectively induce microcephaly and other observed changes in the brain, ZIKV must either alter pathways that affect CNS development or directly infect cells of the CNS. Comparisons to other viral TORCH pathogens strongly support the second possibility. It is worth noting that the early preparation of ZIKV in the laboratory setting was performed by intracerebral passage of the virus in neonatal mice. One study from 1971 presents an excellent microscopic examination of the brains of these mice [172]. The authors catalog disruption of the pyriform cell layer of the Ammon’s horn and an increased number of astrocytes without the presentation of infiltrating leukocytes. Examination of the tissue by electron microscopy reveals infected astroglia and neurons but not microglia. The first indication that this was happening in humans involved histologic and molecular examination of products of conception, including fetal brain tissue, which revealed the presence of viral particles in the brain of a fetus at 32 weeks of gestation [16]. These findings have been supported and confirmed by a second paper examining another infected fetus [15]. These case reports not only support the conclusion that the virus can replicate in cells of the CNS but that the CNS serves as a site of viral persistence long after the mother was exposed. Again, the propensity for first trimester exposures to ZIKV provides clues about the possible mechanisms of neuropathogenesis. During the first trimester, the fetal blood–brain barrier is “leaky” and does not serve as a complete barrier against pathogens. Infection of the placenta in the first trimester and induction of fetal viremia may sufficiently disseminate virus, thereby enabling ZIKV access to the brain. Fetal development of a well-formed blood–brain barrier later in pregnancy may also reduce the risk of CNS infection. A second possibility is that the frequency of target cells in the brain changes over time. A seminal report by Tang et al. reveals that ZIKV can infect neural progenitors [34], and this has been more recently confirmed in a study of ZIKV infection of human cerebral organoids in culture [33,36]. Infection of the brain in the first trimester might lead to infection of these precursor cells and associated pathology because of the ability of ZIKV to slow cellular replication and induce cell death. Supporting this hypothesis, direct examination of tissue from at least one ZIKV-positive fetus indicates that mature neurons are relatively unperturbed, suggesting that the progenitors may be preferentially infected [15]. However, the reports by Bell et al. discussed above as well as recent studies involving a more natural route of infection [173] demonstrate that infection of more mature brain cells is possible [172]. Examination of the literature reveals the presence of Tyro3, AXL, DC-SIGN, and TIM-1 on multiple cells in the CNS, leading to the hypothesis that multiple cell types might be infected (Table 2).

**Table 2:** Expression of ZIKV receptors in human brain and placental tissue. NA = data not available.

	DC-SIGN	AXL	Tyro3	TIM-1	Evidence of Infection	References
**CNS**						
**Vascular Endothelial**	-	+	-	NA	Productive infection in tissue culture	[174–177]
**Perivascular macrophages**	NA	+	+	NA		[178]
**Astroglia**	-	+	+	NA	EM in mice	[174–177,179,180]
**Microglia**	-	+	+	NA		[174–177,181]
**Neurons**	-	+	+	NA	EM in mice	[174–176,180]
**Neuronal Precursors**	NA	NA	NA	NA	Productive infection in tissue culture	[34]
**Placenta**						
**Trophoblast**	-	+	+	NA	Pathology	[170,174]
**Dendritic Cells**	+	+	+	NA		[178,182,183]

Murine models of ZIKV infection have been used to clarify which cells are infected in vivo. Pups born to infected mothers of the SJL strain showed pathology of the CNS similar to that observed in humans, and cortical malformations were observed in the brains of infected pups following birth [70]. This included reduced cell number and thickness of the cortical layer. At the cellular level, neurons in several regions of the brain showed enlarged nuclei because of infection. Gene expression studies using qPCR indicated altered regulation of genes involved in autophagy and apoptosis. A study using direct injection of ZIKV into the brain of embryonic day 13.5 mice showed similar outcomes [158]. In this model, examination of fetal brain tissue 3 to 5 days postinfection revealed infection in the intermediate zone and cortical plate. Thinning of the cortical layer was also observed. Immunostaining of slices from the fetal brain revealed that neural precursor cells were the primary target of the ZIKV. The authors also observed alterations in apoptosis as described by Cugola et al. In addition, a selection of genes previously shown to be involved in microcephaly were determined to be down-regulated in ZIKV infected neural precursors. The results from these 2 mouse models are in agreement with the predictions made from cell culture work. ZIKV preferentially infects neural precursor cells and leads to alterations of the developing brain associated with microcephaly.

### Permissiveness to Viral Infection and Alteration in Cellular Pathways

Not all cells expressing the receptor for a given virus are capable of being productively infected. The presence or absence of specific factors in the cell influence whether the virus can successfully establish an infection and produce more of the virus. At this time, little is known about the intracellular factors that may influence ZIKV replication. It may be that not all cells that display the appropriate receptors are capable of supporting viral replication. Genome-wide RNAi screens have identified hundreds of cellular factors involved in flavivirus replication [184]. Many of these factors are involved in critical host cell pathways, such as the following: nucleic acid production, protein production and transport, lipid metabolism, and energy production [184–186]. Various interferon-responsive genes have been shown to block flavivirus replication, as highlighted by the numerous mechanisms employed by the virus to counter these effects. However, in the absence of interferon, it is unclear if any cells are truly nonpermissive to ZIKV infection.

What is clear is that flaviviruses have evolved multiple strategies for altering normal host cellular pathways to favor viral replication. Stress granules and P-bodies are accumulations of RNA found in the cytoplasm of cells that are involved in stress response, heat shock, and response to infection by viruses [187,188]. Flaviviruses alter both of these granule types to increase viral replication. Interaction of viral noncoding regions with stress granule proteins has been implicated in increased viral RNA synthesis and processing of viral RNA by enzymes in the P-body, which leads to the accumulation of a noncoding viral RNA that may be involved in protecting the viral RNA against RNA interference [189,190].

The existence of flavivirus-encoded noncoding RNA (ncRNA) is of potential relevance to development of fetal neuropathology. The genome of ZIKV and other flaviviruses is relatively small. As such, there is evolutionary pressure to make efficient use of all available sequence to support viral replication and evasion of adaptive and innate host defenses. That the virus supports and maintains RNA and RNA structural motifs that are not directly used in the coding of proteins suggests that this noncoding RNA serves an important role in the viral life cycle [191]. The production of ncRNA in flaviviruses is due to the incomplete digestion of viral RNA by 5'-3' Exoribonuclease 1 (XRN1), an exonuclease found in the P-body [190]. A secondary structure in a stem loop within the untranslated region (UTR) prevents digestion of this area and leads to accumulation of viral ncRNA. Interestingly, this ncRNA seems to be essential for cytopathicity and viral pathogenesis. Viruses with mutations in the 3ʹUTR have no deficit in their ability to make viral RNA but show attenuated cytopathic effects in infected cells. Two possible explanations have been given for this observation. The first is that the ncRNA modulates the host innate sensing proteins (Toll-like receptors including TLR3, RIG-I, and MDA5). Other studies show evidence that this ncRNA can function to inhibit the RNA interference pathway and alter the expression of host genes [192]. When primary human fibroblasts are infected with Dengue virus, innate immune response signaling pathways are activated through both TLR3 and RIG- 1 but not Mda5, triggering up-regulation of human interferon-β

Interferon beta (IFNβ), TNFα, defensin 5 (HB5), and β defensin 2 (HβD2) [193]. Heritable mutations in RIG-I and MDA5 coding sequences have been identified as causative for Type 1 interferonopathies (inherited autoimmune disorders associated with an inborn elevated interferon response), including Aicardi–Goutières syndrome and systemic lupus erythematosus (SLE) in certain individuals as well as classic and atypical Singleton–Merten syndrome [194]. As reviewed above, the radiographic characteristics of these syndromes overlap considerably with findings associated with both intrauterine CMV infection and Zika congenital syndrome. Prior assessment of therapeutic strategies for Aicardi–Goutières syndrome may help inform treatment options for Zika congenital syndrome [195]. Hydroxychloroquine, used to treat SLE cerebritis and considered safe in pregnancy, is a potent inhibitor of Type I IFNs, and this therapeutic strategy may figure into the selection of drug-like entities being contemplated for treating pregnant women suffering from acute ZIKV [196–198].

Interactions of cellular proteins with the untranslated regions of the full-length ZIKV RNA may also be critical for function. Examination of the West Nile virus has shown that two cellular RNA-binding proteins, TIA-1 and TIAR, interact with the 3’UTR of that virus [189,199]. These proteins are essential host factors involved in formation of stress granules and are sequestered at the site of viral RNA synthesis, an event that inhibits stress granule formation [199,200]. Viruses deficient in TIA-1 and TIAR binding replicate at a diminished rate in fibroblasts. A similar mechanism has been described for dengue virus [199]. Because of the similarities to the secondary structure of the 3’UTR of these flaviviruses, ZIKV is likely to have similar effects. Whether ZIKV genomic or subgenomic RNA has binding sites for other host factors remains to be seen. Engagement of RNA-binding proteins specific to the brain or placenta by ZIKV might explain the pathology seen in the current epidemic.

The ability of ZIKV noncoding RNA to recruit cellular proteins might provide some insight into possible mechanisms of neuropathogenesis. The unique sequence of ZIKV may provide new targets for interaction with cellular proteins that are not seen in related viruses such as dengue. Of particular interest will be whether factors specific to either the CNS or placenta bind to and regulate ZIKV RNA translation or replication. For example, the RNA-binding protein Musashi-1 is expressed at high levels in neural precursor cells, and can be found in both decidual and trophoblast cells in the placenta [171,201].

**Fig 2:**
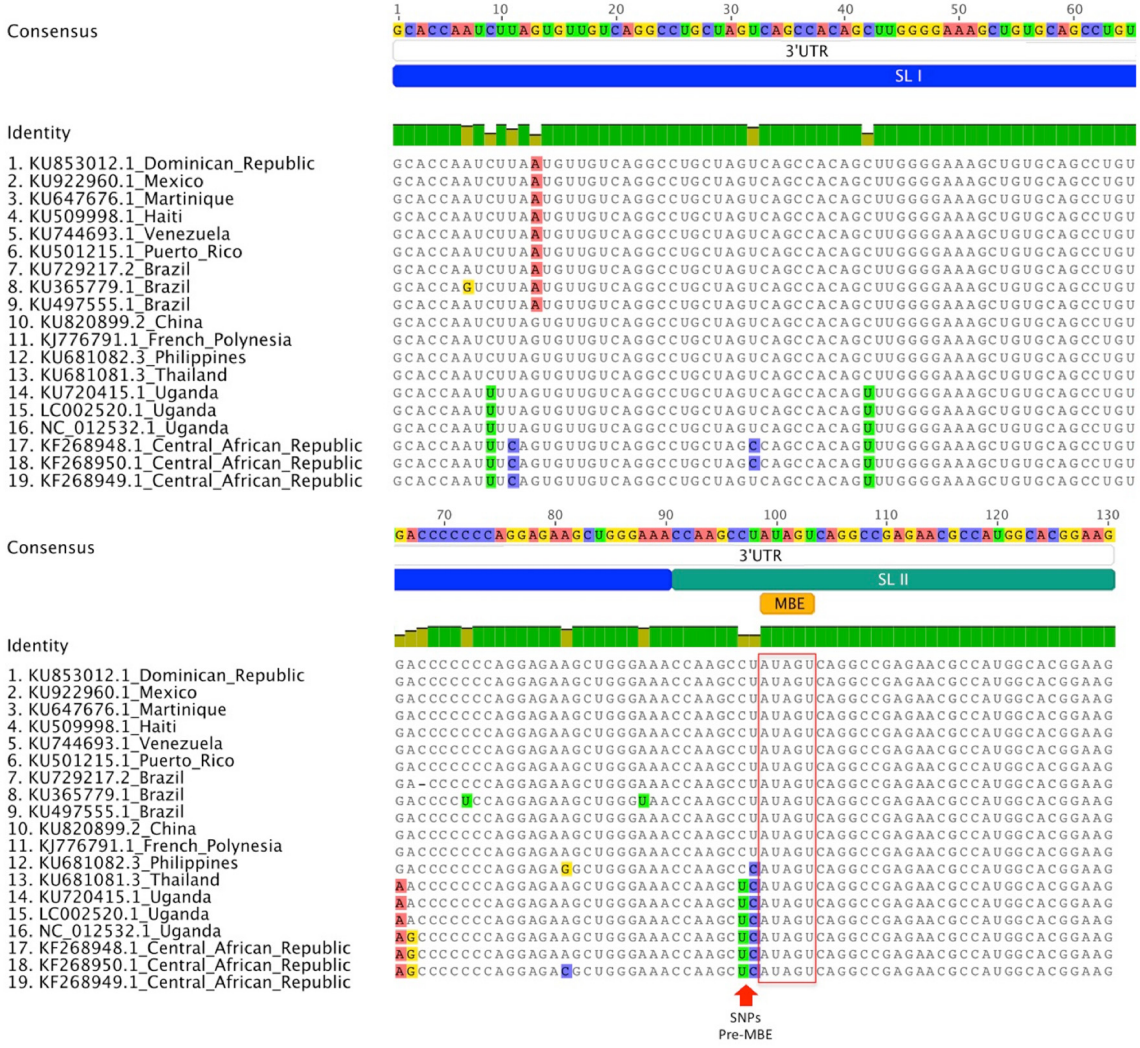
Alignment of first 130 nucleotides of 3’UTR of ZIKV, illustrating Musashi binding element (MBE) location and associated mutations over time and geographic spread. Sequences shown are the only ones that are unique for country and/or sequence; duplicates of the same country were discarded. Alignment was performed using the MAFFT multiple sequence alignment program for unix-like operating systems. Visualization was performed using Geneious. There is presence of stem-loop I (SL I) and stem-loop II (SL II) on those sequences, with SL II being partially shown. There is also presence of MBE on SL II, with two SNPs on African sequences, which could potentially change the RNA structure and availability of the element. SL I and SL II were annotated from Zhu Z. et al. MBE was annotated using the UTRscan tool of UTRSite (http://utrsite.ba.itb.cnr.it/).

Musashi-1 is required for differentiation and division of neural precursors and is often used as a marker in identification of these cells [202,203]. Studies have revealed a role for Musashi as a regulator of mRNA translation and that the protein is capable of both inhibiting and activating translation [204]. Specifically, Musashi proteins play a role in regulating progenitor (stem) cell growth and differentiation through posttranscriptional control of gene expression [205]. Musashi is also expressed in—and has been shown to influence mRNA translation in—a variety of epithelial stem cell types associated with glandular epithelium [205–208], spermatogenesis [209], and brain and retinal tissue development [210,211]. Utilizing sequence alignment methods and available genomes of both historic and current ZIKV isolates, we have discovered a putative Musashi binding element (MBE) in the SL2 stem-loop of the 3’UTR (Fig 2) [212–215]. Examination of ZIKV epidemic strains has revealed conserved changes in the NS2B open reading frame and 3’UTR relative to ancestral strains found in Africa [215]. Our alignment confirms this and highlights that two of these changes lie immediately upstream from the putative MBE. Both insects and mammals have Musashi homologs, and it has been reported that they bind MBE with slightly different sequence requirements [216]. Application of the binding energy predictions of this work suggests that the evolutionary nucleotide polymorphism alterations observed in the region immediately upstream to the ZIKV core MBE may alter binding in mammals but not the mosquito host. Given the expression of Musashi in neuronal precursors and the placenta, it will be critical to determine whether this element is involved in ZIKV pathogenesis and, if so, what ZIKV nucleotide polymorphisms may be associated with alterations in ZIKV MBE activity. The demonstration of qualitative reduction of proliferative cell migration from Mushashi-positive cells after ZIKV infection of human neurospheres is consistent with the hypothesis that the MBE present in the 3’UTR of ZIKV may play a functional role in controlling ZIKV replication in these cell types [70].

Flavivirus proteins insert themselves into the membrane of the endoplasmic reticulum (ER), forming invaginations that contain all of the proteins and RNA needed to produce additional viral RNA [217]. These invaginations are connected to the cytoplasm by a small pore, through which the RNA is presumably passed to engage nearby ribosomes [218]. Viral capsids are then assembled and enveloped by budding into the membranes of the Golgi. This dependence on membranes and the need to produce enough phospholipids to coat all of the progeny virions has lead flaviviruses to evolve mechanisms to alter membrane synthesis, lipid metabolism, and ER processing [219–224].

The classic sign of flavivirus infection is the visualization by electron microscopy of small “viral factories” where viral RNA and protein is made and then assembled into complete virions for release through the cellular transport system. It has been noted that these assemblages look very much like the autophagosomes formed during the process of autophagy. Autophagy is a normal cellular process wherein the cell digests large protein complexes or intracellular pathogens and has been shown to play an important role in the maintenance of stem cells [225]. This process can provide a way for a cell to recycle materials under conditions of starvation or as a way to respond to intracellular infection [226]. Studies of cells infected by ZIKV and other flaviviruses have shown an increase in the levels of autophagy [168,227–229]. Microscopic examination of intracellular compartments has revealed the presence of viral envelope protein (E protein) in the same vesicles as the autophagy marker LC3 [168]. This suggests that the vesicles into which the virus buds may be autophagosomes. Some viruses block the late stages of autophagy, leading to the accumulation of autophagosomes that do not fuse with the lysosome. However, it seems that ZIKV does not block this step, and LC3 and E protein can be detected in mature autolysosomes. As the proper maturation of the viral envelope prior to release is pH dependent, it is possible that the virus has coopted this pathway to maintain the correct pH and access proteases needed for maturation of the viral E protein. The trophoblast layer of the placenta produces microRNA (miRNA) that are pro-autophagic in nature and are delivered to bystander cells by exosomes [144]. It is thought that this is a mechanism to make the trophoblast (and the cells in contact with it) more resistant to viral infection. However, in the case of ZIKV, this mechanism may help replication and spread by the virus once initial infection has been established and could increase the susceptibility of nearby myeloid cells. Multiple lines of research suggest a role for autophagy in neurodegenerative diseases, which indicates that these ZIKV-mediated changes in autophagy may also be involved in the observed neuropathic effects [226,230,231]. Pharmacologic inhibition of autophagy is associated with inhibition of ZIKV replication in a variety of cell types, including human astrocytes [7,168].

Although relatively little work has been done to describe the mechanisms behind microcephaly caused by infectious agents, inherited microcephaly has been fairly well studied. Next-generation sequencing studies have identified a number of genes involved in primary autosomal recessive microcephaly [232]. These studies have revealed common targets relating to cell cycle progression and specifically to mitotic spindle formation [233]. Although it has been suggested that studies should examine these genes in ZIKV and other TORCH pathogens [234], no data demonstrating that ZIKV specifically alters the function of mitotic spindle proteins in infected CNS cells are currently reported.

## Unanswered Questions

In order to more completely understand the link between ZIKV infection and fetal abnormalities, more work must be done. The characteristic presentation of Zika congenital syndrome ranges from viral centric (microcephaly, blindness, ventricular calcifications, and fetal presence of ZIKV by rt-PCR) to another extreme (long bone dysgenesis, negative for ZIKV) possibly associated with placental insufficiency. Epidemiological assessment of potential confounding risk factors for Zika congenital syndrome—including preceding immunologically cross-reactive arboviral infection [7,18,19]—and potential thalidomide sharing by patients being treated for leprosy [27–30] remains to be completed. To underscore the point, leprosy is now endemic throughout much of Brazil, including Pernambuco [235], and postexposure prophylaxis of exposed individuals has been advocated [236,237]. The possibility of intrauterine exposure to pyriproxifen-containing insecticides (in northeastern Brazil) with subsequent teratogenesis because of activation of retinoid X receptors has been repeatedly raised [26], and resolution of this controversy may require an appropriately powered epidemiological exposure study.

The gaps in understanding of ZIKV neuropathology highlighted in this review suggest that efforts should first be focused on obtaining clear, statistically significant data addressing a few specific questions. Prospective case control study reports on ZIKV infection of pregnant women and fetal outcomes are a step in the right direction. As such studies continue, a more definitive correlation between ZIKV infection and various congenital outcomes will become possible. Additionally, fundamental research will be required to answer questions regarding the ability of ZIKV to cross the placenta and infect the developing brain. Based on the published report of receptors utilized by ZIKV, a more complete survey of expression levels of these proteins in cells of the placenta should be prioritized. There is a desperate need for high quality histology and EM analysis of brain and placental tissue from different times after exposure. Although the Mlakar et al. report showed convincing evidence for the presence of viral particles in the brain of a 32-week fetus, the method of fixation unfortunately makes it impossible to tell what specific cells may have been infected [16]. A more recent analysis provides better clarity, but more studies will be needed [15]. Some conclusions may be inferred from the work of Bell et al., but the injection of virus directly into the brain of neonatal mice may not be physiologically relevant [172]. Recent progress involving the development and characterization of ZIKV infection using the AG129 mouse model are consistent with the findings of Bell et al. and may eventually enable a more complete understanding of the neural and glial tropism underlying ZIKV neuropathology [173]. Although current literature provides some characterization of placental abnormalities, no definitive evidence has been shown supporting infection of specific cells of the placenta. A well-characterized animal model likely will be required to obtain this data, preferably one with a more intact interferon response [154–156]. Finally, although PCR and histology are potentially powerful techniques, definitive proof of infection of a given tissue or the relevance of a virus reported in a biological sample can only be obtained when replication-competent viruses can be retrieved from these samples.

To begin to understand the mechanism of ZIKV neuropathogenesis, other experiments might be considered. A survey of serum from ZIKV-infected individuals could shed light on the development of self-reactive antibodies and possible links to GBS. Prior research and study designs that have illuminated the roles of viral proteins and regions or motifs of viral RNA in the pathogenesis of other flavivirus infections need to be applied to clarify the molecular virology of ZIKV. To what extent does ZIKV activation of TLR-3 contribute to fetal neuropathology? Are migratory placental cells such as Hofbauer cells actually infected by ZIKV during fetal development? Do specific proteins from the placenta and brain bind to the noncoding regions of ZIKV and play a role in the observed neural tissue disease? Recent studies have cataloged changes in the ZIKV genome as it has spread across the Pacific to the new world. Specific studies will be necessary to determine if these changes have in any way altered the transmissibility or virulence of the virus. Finally, the studied TORCH pathogens do not consistently cause pathology. It has been hypothesized that ZIKV infection may achieve access to the placenta and CNS secondary to some other event. Larger datasets will be needed to determine if ZIKV enters the fetus following some other perturbation or whether other cofactors or confounding variables are associated with the severe congenital and adult neuropathology, which is now being observed with the current ZIKV outbreak in the Americas. But what is most clear is that ZIKV fetal neuropathology represents a new disease which does not completely overlap with the epidemiology or pathophysiology of other TORCH pathogens and which will demand effort, resources, unparalleled collaboration, and, above all, open-mindedness in formulating public health responses as well as obstetrical and pediatric management strategies.

**Key Learning Points**

- Viral TORCH pathogens reveal common patterns of fetal pathophysiology and vertical transmission, which have histopathologic correlates in Zika virus fetal neuropathogenesis.
- The teratogenic effects of Zika virus infection during the first trimester may involve infection of the trophoblast, viral translocation across the placenta, migration of infected cells resulting in embryonic infection, or indirect effects associated with high levels of inflammatory cytokines produced by infected placental tissues.
- Zika virus activation of Toll Like Receptor 3 (TLR-3) pathways in central nervous system cells may trigger apoptosis and attenuate neurogenesis, directly contributing to fetal neuropathology.
- Recognition of viral sequences by regulatory RNA-binding proteins such as Musashi may have a role in Zika pathogenesis and host tissue tropism.
- Evidence from other TORCH viral pathogen studies indicate multiple plausible hypotheses for transplacental infection by Zika virus during the second or third trimester, including transcytosis of non-neutralizing antibody-coated Zika virus complexes; preexisting maternal non-neutralizing antibody to Zika or other flaviviruses may enhance the probability of infection or more severe disease in the fetus.

**Top Five Papers**

1. Adibi JJ, Marques ET Jr, Cartus A, Beigi RH. Teratogenic effects of the Zika virus and the role of the placenta. Lancet 2016; 387: 1587–90 (Hypothesis).
2. Mlakar J, Korva M, Tul N, Popović M, Poljšak-Prijatelj M, Mraz J, et al. Zika Virus Associated with Microcephaly. N Engl J Med. 2016;374:951–958.
3. Tonduti D, Orcesi S, Jenkinson EM, Dorboz I, Renaldo F, Panteghini C, et al. Clinical, radiological and possible pathological overlap of cystic leukoencephalopathy without megalencephaly and Aicardi-Goutieres syndrome. Eur J Paediatr Neurol. 2016;20(4):604–10.
4. Dang J, Tiwari SK, Lichinchi G, Qin Y, Patil VS, Eroshkin AM, Rana TM. Zika Virus Depletes Neural Progenitors in Human Cerebral Organoids through Activation of the Innate Immune Receptor TLR3. Cell Stem Cell. 2016:19: 1–8.
5. Cugola FR, Fernandes IR, Russo FB, Freitas BC, Dias JLM, Guimarães KP, Benazzato C, Almeida N, Pignatari GC, Romero S, Polonio CM, Cunha I, Freitas CL, Brandão WN, Rossato C, Andrade DG, Faria DP, Garcez AT, Buchpigel CA, Braconi CT, Mendes E, Sall AA, Zanotto PMA, Peron JPS, Muotri AR, Beltrão-Braga PCB. The Brazilian Zika virus strain causes birth defects in experimental models. Nature. 2016;534(7606):267–71.
